# Supplementary material for: HIV status alters immune cell infiltration and activation profile in women with breast cancer
Source: Nat Commun. 2025 May 20;16:4699. doi: 10.1038/s41467-025-59408-8 (PMC12092689; doi:10.1038/s41467-025-59408-8)
Supplement: Supplementary file 1 — Supplementary Information [file 41467_2025_59408_MOESM1_ESM.pdf]

Supplementary Files

HIV status alters immune cell infiltration and activation profile in women with breast cancer

Marcus Bauer[1,2*], Pablo Santos[2], Andreas Wilfer[1,3], Eunice van den Berg[4], Annelle Zietsman[5], Martina Vetter[6], Sandy Kaufhold[6], Claudia Wickenhauser[1], Isabel dos-Santos-Silva[7], Wenlong Carl Chen[8,9,10], Herbert Cubasch[11], Nivashini Murugan[11], Valerie McCormack[12], Maureen Joffe[8,13], Barbara Seliger[14,15,16*], Eva Kantelhardt[2,6]

*First author and corresponding author

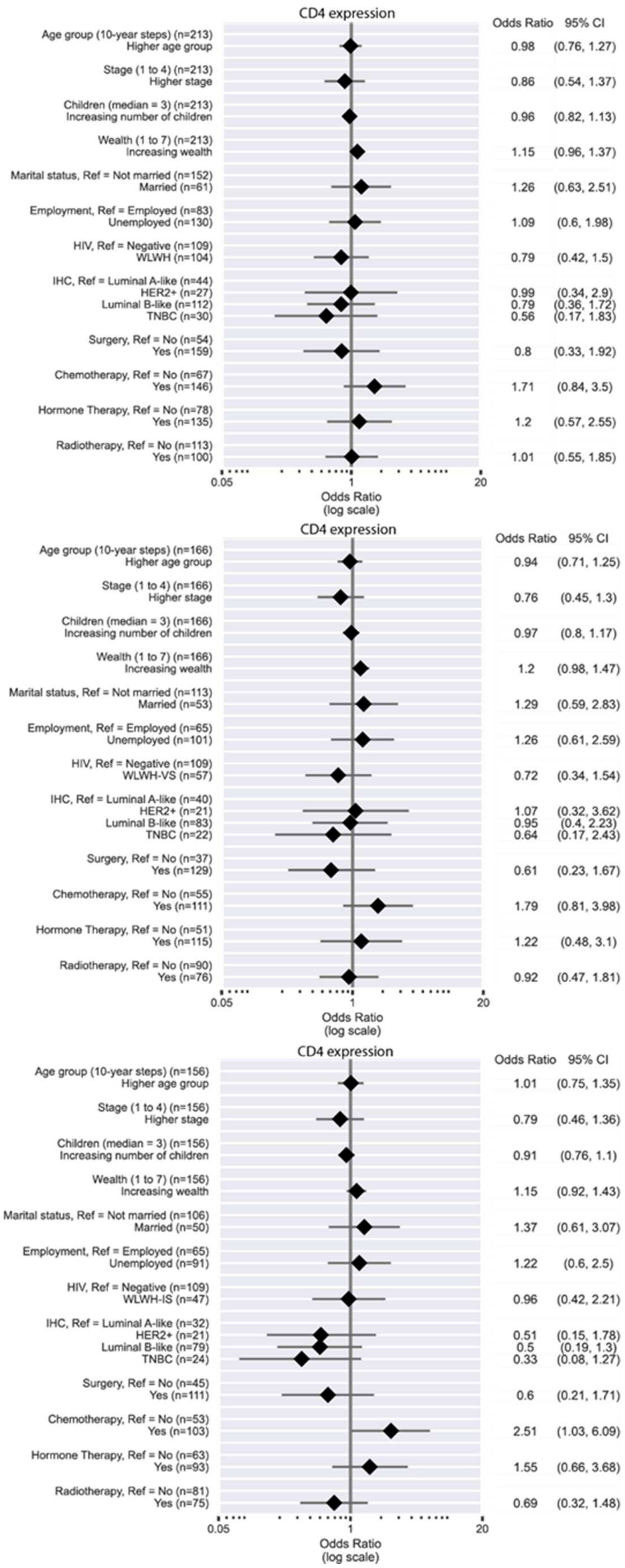

Supplementary Figure 1: HIV and CD4 mRNA expression. Association between HIV status and CD4 RNA expression in WLWH - VS and WLWH - IS versus HIV negative BC patients in a multivariate analysis, depicted as a forest plot.

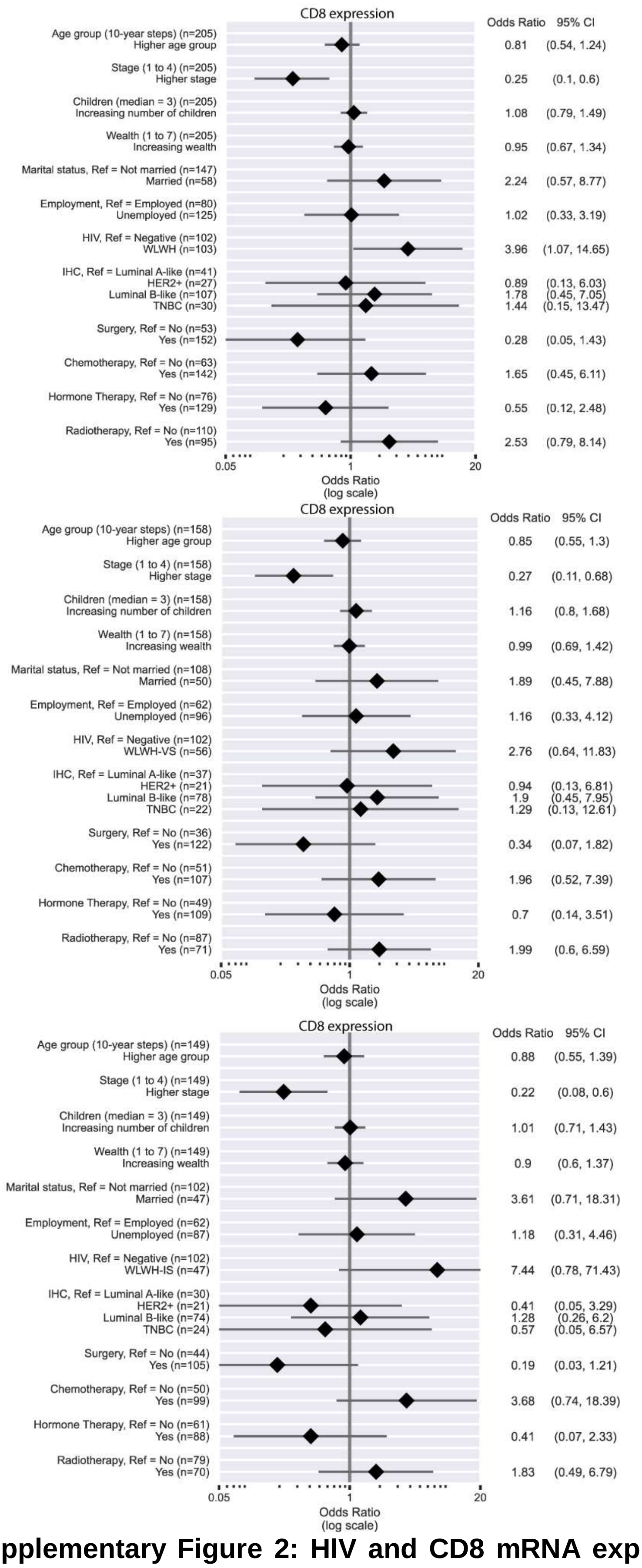

Supplementary Figure 2: HIV and CD8 mRNA expression. Association between HIV status and CD8 RNA expression in WLWH - VS and WLWH - IS versus HIV negative BC patients in a multivariate analysis, depicted as a forest plot.

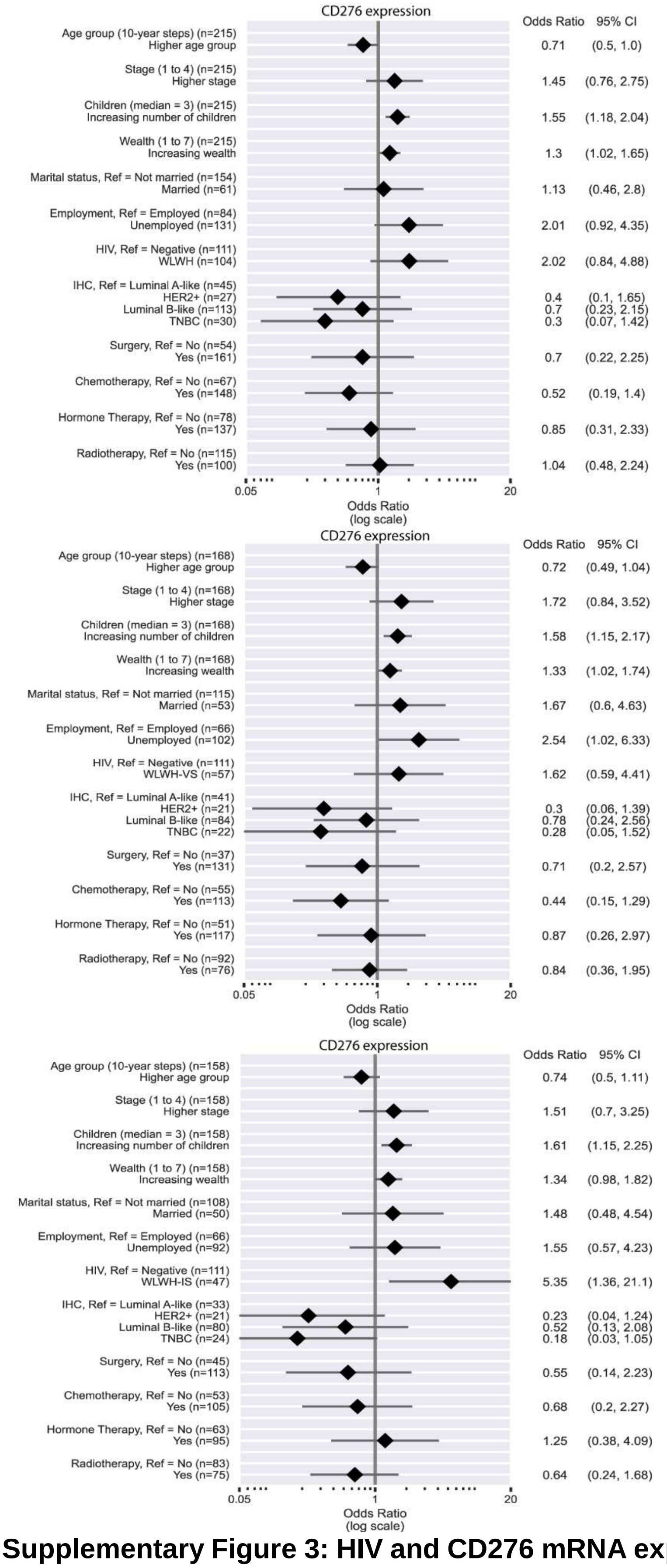

Supplementary Figure 3: HIV and CD276 mRNA expression. Association between HIV status and CD276 RNA expression in WLWH - VS and WLWH - IS versus HIV negative BC patients in a multivariate analysis, depicted as a forest plot.

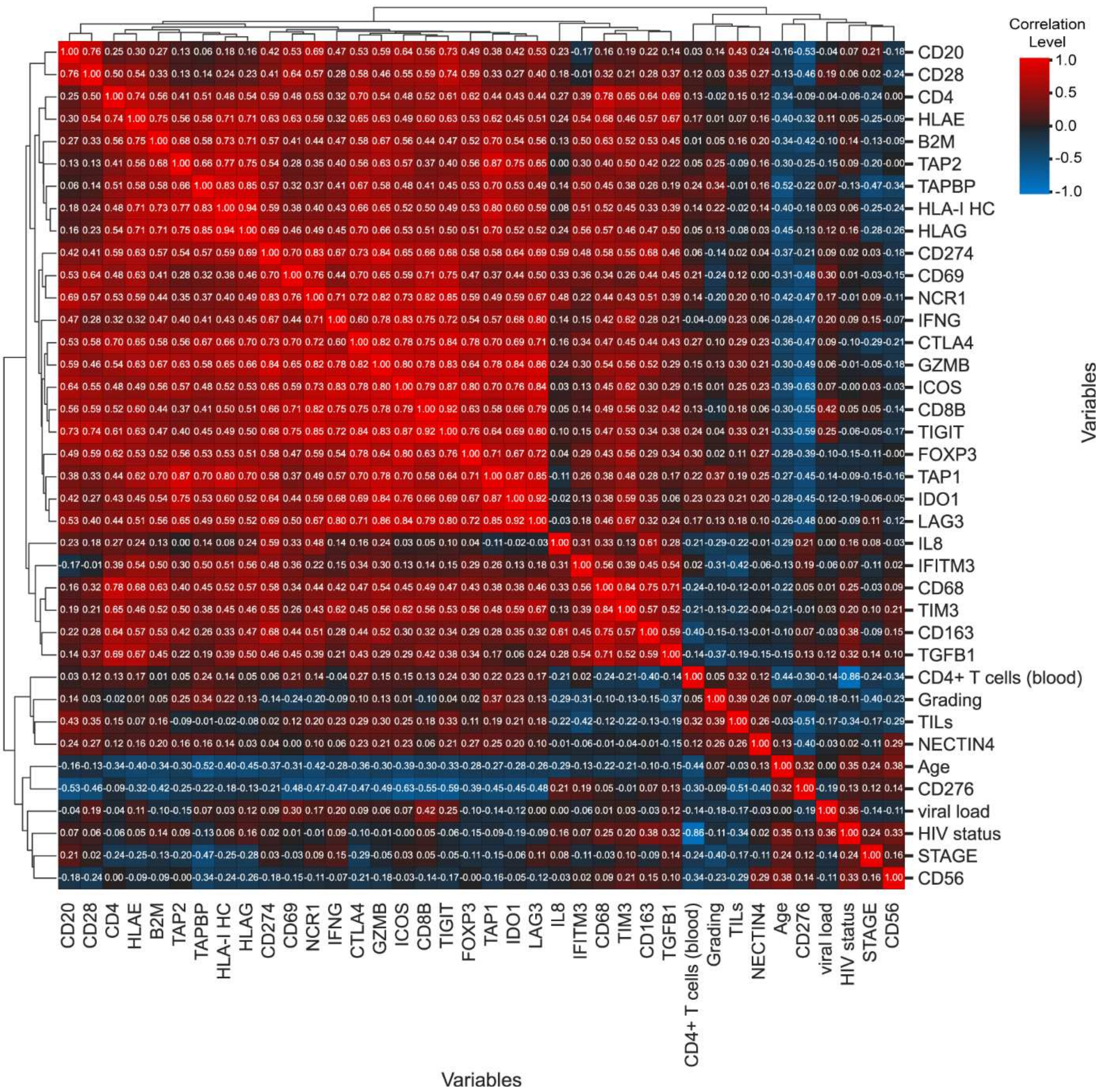

Supplementary Figure 4: Correlation of factors in the TME including all BC samples. Correlation map of selected immune genes in all patients, irrespective of the HIV status. The correlation values are shown in different colors with red tiles denote a positive correlation, while blue tiles correspond to a negative correlation.

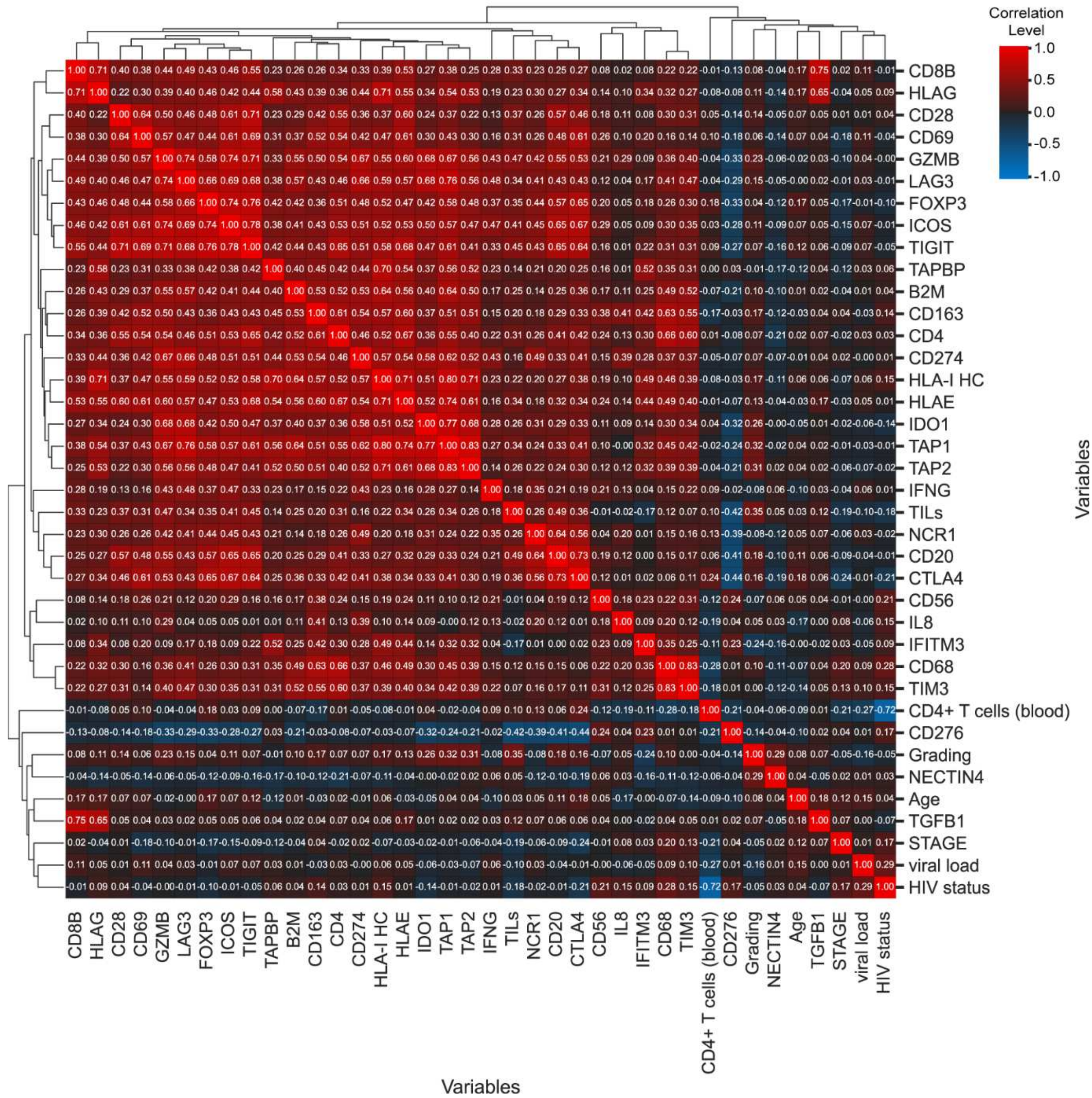

Supplementary Figure 5: Correlation of factors in the TME of WLWH. Correlation map of selected immune genes in WLWH. The correlation values are shown in different colors with red tiles denote a positive correlation, while blue tiles correspond to a negative correlation.

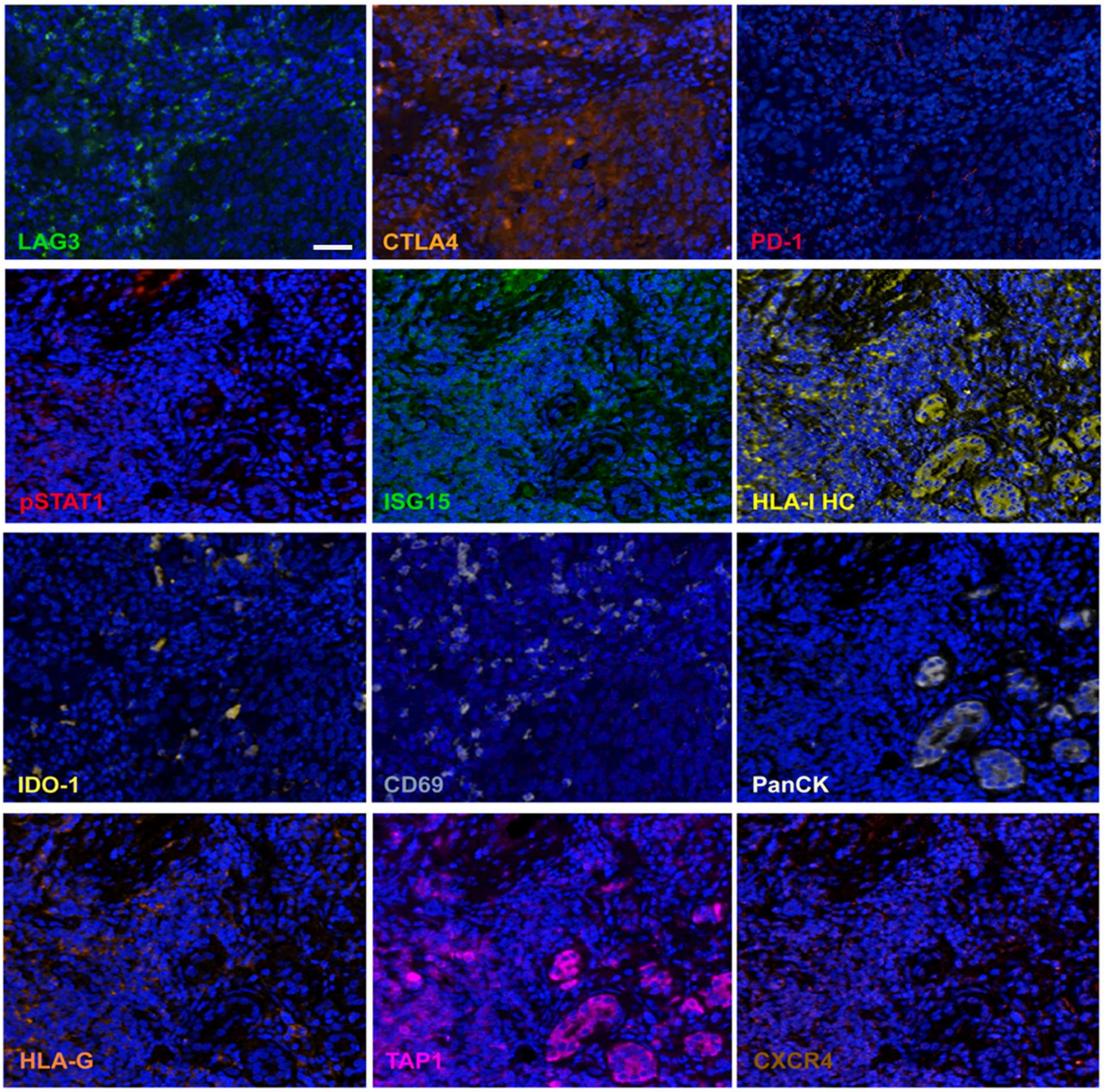

Supplementary Figure 6: Multispectral imaging panel. Representative pictures of staining for LAG3 (green), CTLA4 (orange), PD-1 (red), IDO-1 (yellow), CD69 (white), components of the IFN-γ signaling pathway including pStat1 (red), ISG15 (green) as well as for HLA-I HC (yellow), HLA-G (orange), TAP1 (magenta), CXCR4 (brown) and panCK$^+$ cancer cells (grey). The scale bar depicts 50 µm.

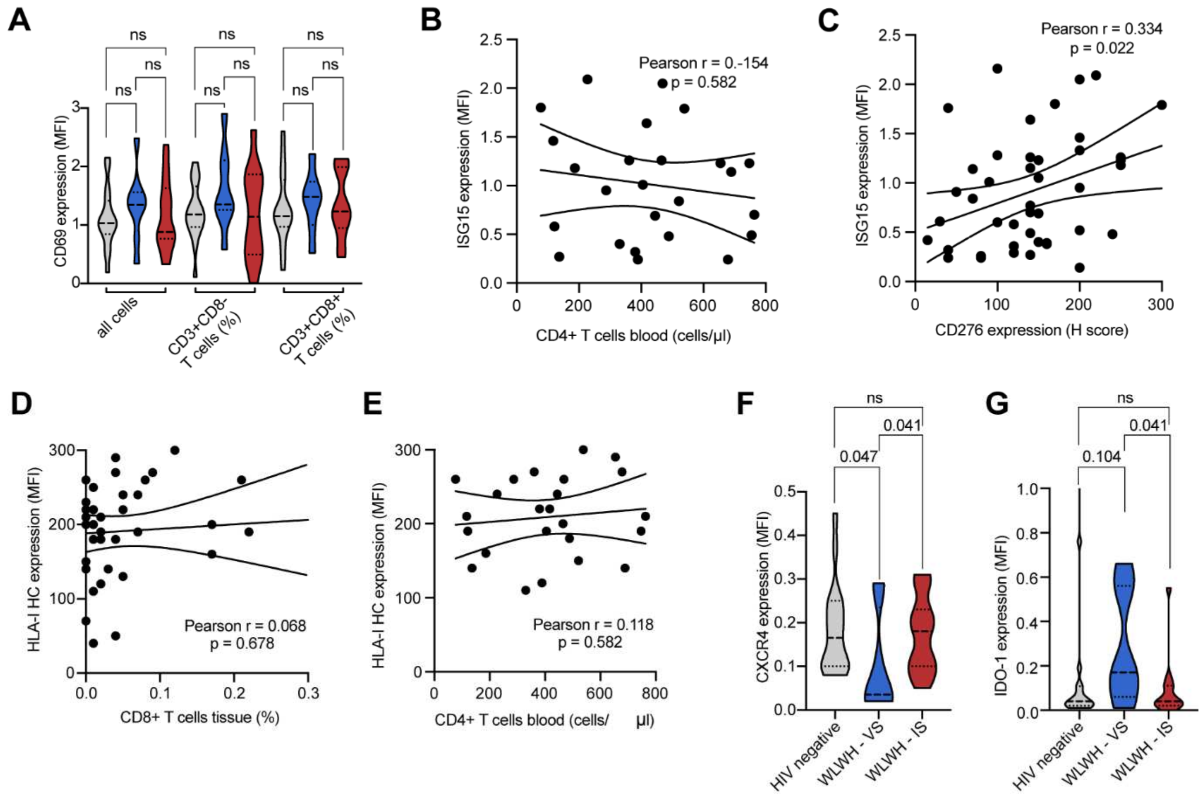

Supplementary Figure 7: Expression of immune-response relevant molecules in the BC TME. (A) The expression of CD69 on different cell populations in WLWH and HIV negative BC patients. The mean is given as a dashed line and the standard deviation is given as a dot line. Categories were compared using the Mann–Whitney U test. (B-C) Scatter plot depiction of ISG15 expression with CD4^{+} T cells in the peripheral blood and CD276 expression in the BC tissue. The graph is overlaid with the corresponding linear regression model and associated p-values (two-sided) are given. (D-E) Scatter plot depiction of the interrelationship of HLA-I HC expression and CD3^{+}CD8^{+} T cells or CD4^{+} T cells in the peripheral blood. The graph is overlaid with the corresponding linear regression model and associated p-values (two-sided) are given. (F-G) Distribution of CXCR4 and IDO-1 expression in tumor specimens of WLWH and HIV negative BC patients. The mean is given as a dashed line and the standard deviation is given as a dot line. Categories were compared using the Mann–Whitney U test and p-values are given as numbers if $p < 0.05$.

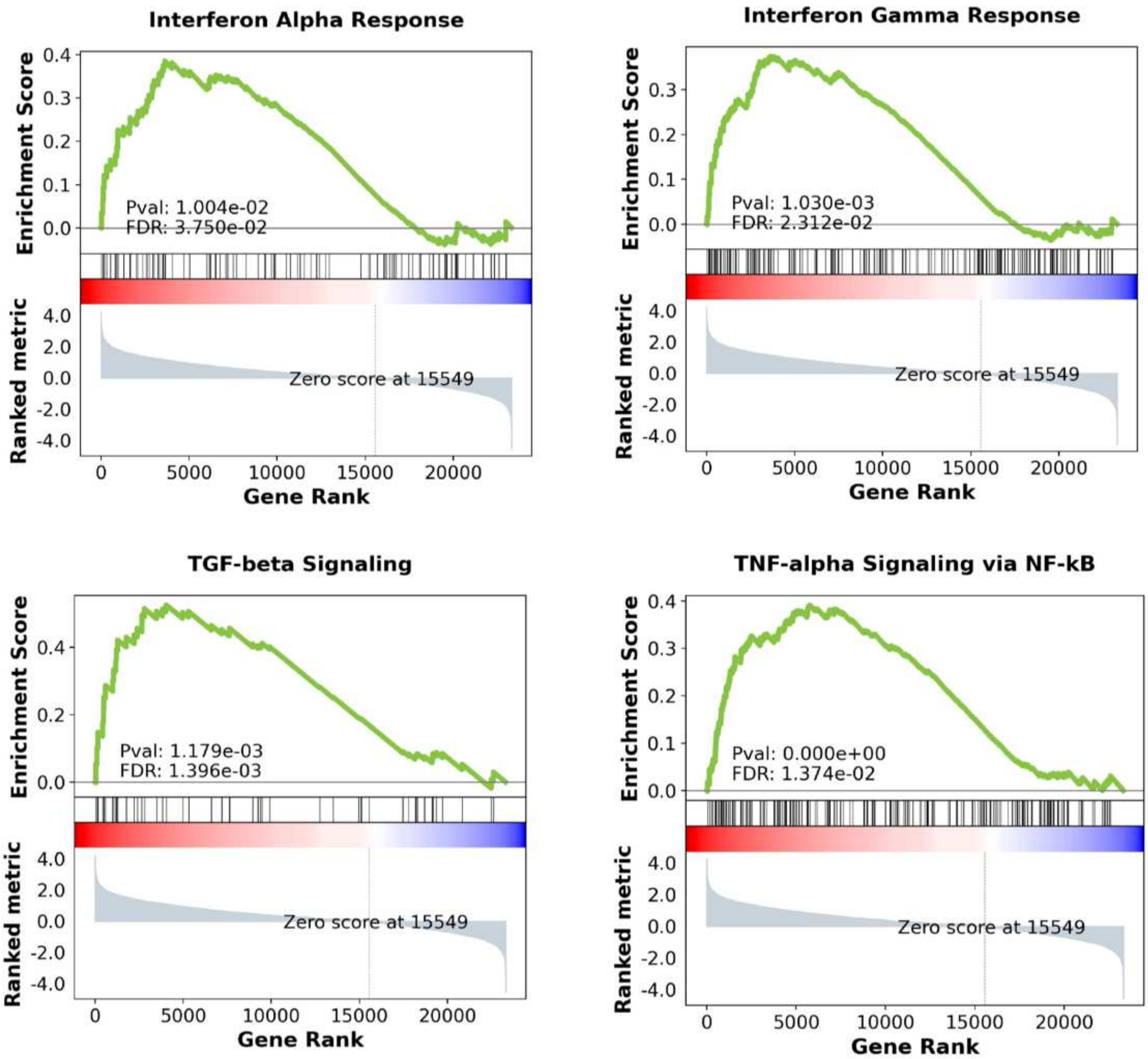

Supplementary Figure 8: GSEA of the gene expression in the BC TME of WLWH versus HIV negative BC patients. GSEA plots of immune pathways (MSigDB_Hallmark_2020) using GSE149156 including specimens from WLWH versus HIV negative BC patients. Abbreviations: GSEA, gene set enrichment analysis. Abbreviations: GSEA, gene set enrichment analysis

Supplementary Table 1: Genes and NCBI accession codes used for the RNA expression analysis

Gene name	Reference sequence	Oligonucleotide sequence
B2M	NM_004048.2:235	TACTGAAGAATGGAGAGAGAATTGAAAAAGTGGAGCATTCAGACTTGTCTTTCAGCAAGGACTGGTCTTTCTATCTCTTGTACTACACTGAATTCACCCC
BDCA-2	NM_001371390	GCCTCGCTGCTTTCTTTTCTCCAAGACGGGCTGAGGATTGTACAGCTCTAGGCGGAGTTGGGGCTCTTCGGATCGCTTAGATTCTCCTCTTTGCTGCATT
CCR2	NM_001123041.2:743	TCTGATCTGCTTTTTCTTATTACTCTCCCATTGTGGGCTCACTCTGCTGCAAATGAGTGGGTCTTTGGGAATGCAATGTGCAAATTATTCACAGGGCTGT
CD11c	NM_000887.4	TGAGGGTACGGAGACCACAAGCAGTAGCTCCTTCGAATTGGAGATGGCACAGGAGGGCTTCAGCGCTGTGTTCACACCTGATGGCCCCGTTCTGGGGGCT
CD163	NM_004244.4:1630	CATCTGTGATTCGGACTTCTCTCTGGAAGCTGCCAGCGTTCTATGCAGGGAATTACAGTGTGGCACAGTTGTCTCTATCCTGGGGGGAGCTCACTTTGGA
CD19	XM_011545981.1:713	CACCCCAAGGGGCCTAAGTCATTGCTGAGCCTAGAGCTGAAGGACGATCGCCCGGCCAGAGATATGTGGGTAATGGAGACGGGTCTGTTGTTGCCCCGGG
CD2	NM_001767.2:1400	TGGGTCTCACTACAAGCAGCCTATCTGCTTAAGAGACTCTGGAGTTTCTTATGTGCCCTGGTGGACACTTGCCCACCATCCTGTGAGTAAAAGTGAAATA
CD20	NM_152866.2	CTTCTGATGATCCCAGCAGGGATCTATGCACCCATCTGTGTGACTGTGTGGTACCCTCTCTGGGGAGGCATTATGTATATTATTTCCGGATCACTCCTGG
CD21	NM_001006658.1	GGTGTCAAGCAAATAATATGTGGGGGCCGACACGACTACCAACCTGTGTAAGTGTTTTCCCTCTCGAGTGTCCAGCACTTCCTATGATCCACAATGGACA
CD274	NM_014143.3:49	AGCTTCCCGAGGCTCCGCACCAGCCGCGCTTCTGTCCGCCTGCAGGGCATTCCAGAAAGATGAGGATATTTGCTGTCTTTATATTCATGACCTACTGGCA
CD276	NM_001024736.1:2120	ACATTTCTTAGGGACACAGTACACTGACCACATCACCACCCTCTTCTTCCAGTGCTGCGTGGACCATCTGGCTGCCTTTTTTCTCCAAAAGATGCAATAT
CD28	NM_001243078.1:2065	GCTGCTCCTGTACCTTGGAGGTCCATTCACATGGGAAAGTATTTTGGAATGTGTCTTTTGAAGAGAGCATCAGAGTTCTTAAGGGACTGGGTAAGGCCTG
CD4	NM_000616.4:975	TGGCAGGCGGAGAGGGCTTCCTCCTCCAAGTCTTGGATCACCTTTGACCTGAAGAACAAGGAAGTGTCTGTAAAACGGGTTACCCAGGACCCTAAGCTCC
CD56	NM_000615.5	GGTATTTGCCTATCCCAGTGCCACGATCTCATGGTTTCGGGATGGCCAGCTGCTGCCAAGCTCCAATTACAGCAATATCAAGATCTACAACACCCCCTCT
CD68	NM_001251.2:1140	ACCGGTCCATCTTGCTGCCTCTCATCATCGGCCTGATCCTTCTTGGCCTCCTCGCCCTGGTGCTTATTGCTTTCTGCATCATCCGGAGACGCCCATCCGC
CD69	NM_001781.1:460	AGGACATGAACTTTCTAAAACGATACGCAGGTAGAGAGGAACACTGGGTTGGACTGAAAAAGGAACCTGGTCACCCATGGAAGTGGTCAAATGGCAAAGA
CD8B	NM_172099.2	TCAGCTGAGTGTGGTTGATTTCCTTCCCACCACTGCCCAGCCCACCAAGAAGTCCACCCTCAAGAAGAGAGTGTGCCGGTTACCCAGGCCAGAGACCCAG
CTLA4	NM_005214.3:405	AGTCTGTGCGGCAACCTACATGATGGGGAATGAGTTGACCTTCCTAGATGATTCCATCTGCACGGGCACCTCCAGTGGAAATCAAGTGAACCTCACTATC
CXCR6	NM_006564.1:95	TTACCATGAAGACTATGGGTTCAGCAGTTTCAATGACAGCAGCCAGGAGGAGCATCAAGACTTCCTGCAGTTCAGCAAGGTCTTTCTGCCCTGCATGTAC
FLT3LG	NM_001459.3:350	CCTCCCCAGAATGGAGGCAACGCCAGAATCCAGCACCGGCCCCATTTACCCAACTCTGTACAAAGCCCTTGTCCCCATGAAATTGTATATAAATCATCCT
FOXP3	NM_014009.3:1230	GGGCCATCCTGGAGGCTCCAGAGAAGCAGCGGACACTCAATGAGATCTACCACTGGTTCACACGCATGTTTGCCTTCTTCAGAAACCATCCTGCCACCTG
FURIN	NM_002569.2:3700	GGGTCCCAGTGGGAGGGGCAGGCTGACATCTGTGTTTCAAGTGGGGCTCGCCATGCCGGGGGTTCATAGGTCACTGGCTCTCCAAGTGCCAGAGGTGGGC
GZMB	NM_004131.3:540	ACACTACAAGAGGTGAAGATGACAGTGCAGGAAGATCGAAAGTGCGAATCTGACTTACGCCATTATTACGACAGTACCATTGAGTTGTGCGTGGGGGACC
HLA-I	NM_002116.5:1000	GGAAGAGCTCAGATAGAAAAGGAGGGAGTTACACTCAGGCTGCAAGCAGTGACAGTGCCCAGGGCTCTGATGTGTCCCTCACAGCTTGTAAAGTGTGAGA
HLA-E	NM_005516.4:1204	TGTCTTAGGGGACTCTGGCTTCTCTTTTTGCAAGGGCCTCTGAATCTGTCTGTGTCCCTGTTAGCACAATGTGAGGAGGTAGAGAAACAGTCCACCTCTG
HLA-G	NM_002127.5:289	CGGCCCGGCCGCGGGGAGCCCCGCTTCATCGCCATGGGCTACGTGGACGACACGCAGTTCGTGCGGTTCGACAGCGACTCGGCGTGTCCGAGGATGGAGC
ICOS	NM_012092.2:640	AACTCTGGCACCCAGGCATGAAGCACGTTGGCCAGTTTTCCTCAACTTGAAGTGCAAGATTCTCTTATTTCCGGGACCACGGAGAGTCTGACTTAACTAC
IDO1	NM_002164.5:369	CTATTATAAGATGCTCTGAAAACTCTTCAGACACTGAGGGGCACCAGAGGAGCAGACTACAAGAATGGCACACGCTATGGAAAACTCCTGGACAATCAGT
IFITM3	NM_021034.2:477	TGCTGATCTTCCAGGCCTATGGATAGATCAGGAGGCATCACTGAGGCCAGGAGCTCTGCCCATGACCTGTATCCCACGTACTCCAACTTCCATTCCTCGC
IFNG	NM_000619.2:970	ATACTATCCAGTTACTGCCGGTTTGAAAATATGCCTGCAATCTGAGCCAGTGCTTTAATGGCATGTCAGACAGAACTTGAATGTGTCAGGTGACCCTGAT
IL8	NM_000584.2	ACAGCAGAGCACACAAGCTTCTAGGACAAGAGCCAGGAAGAAACCACCGGAAGGAACCATCTCACTGTGTGTAAACATGACTTCCAAGCTGGCCGTGGCT
LAG3	NM_002286.5:1735	CTTTTGGTGACTGGAGCCTTTGGCTTTCACCTTTGGAGAAGACAGTGGCGACCAAGACGATTTTCTGCCTTAGAGCAAGGGATTCACCCTCCGCAGGCTC
NCR1	NM_004829.5:602	CGATGTTTTGGCTCCTATAACAACCATGCCTGGTCTTTCCCCAGTGAGCCAGTGAAGCTCCTGGTCACAGGCGACATTGAGAACACCAGCCTTGCACCTG
NECTIN4	NM_030916.2:856	GGTCAAAGGCACAACGTCCAGCCGTTCCTTCAAGCACTCCCGCTCTGCTGCCGTCACCTCAGAGTTCCACTTGGTGCCTAGCCGCAGCATGAATGGGCAG
PRF1	NM_005041.3:2120	ACTGTTTTTCAGGGAGGTGGCTGGGTTTACACGCTAATCCCGATTCACCCTGTCCAAACTGCCTAAGCCCTCCGCCATTCTCAAGCCCTGCAGTCACAGC
TAP1	NM_000593.5:2075	GTGGCTGCAGTGGGACAAGAGCCACAGGTATTTGGAAGAAGTCTTCAAGAAAATATTGCCTATGGCCTGACCCAGAAGCCAACTATGGAGGAAATCACAG
TAP2	NM_000544.3:909	GGCTTCCTTTAAATGCCAATGTGCTCTTGCGAAGCCTGGTGAAAGTGGTGGGGCTGTATGGCTTCATGCTCAGCATATCGCCTCGACTCACCCTCCTTTC
TAPBP	NM_003190.4:1082	CATGGGGCCCATGGACCGGAAATGGGACCTTCTGGCTGCCTACAGTTCAACCCTTTCAGGAGGGCACCTATCTGGCCACCATACACCTGCCATACCTGCA
TGFB1	NM_000660.3:1260	TATATGTTCTTCAACACATCAGAGCTCCGAGAAGCGGTACCTGAACCCGTGTTGCTCTCCCGGGCAGAGCTGCGTCTGCTGAGGCTCAAGTTAAAAGTGG
TIGIT	NM_173799.2:1968	TGGATCTTAGAAGACTTTTATCCTTCCACCATCTCTCTCAGAGGAATGAGCGGGGAGGTTGGATTTACTGGTGACTGATTTTCTTTCATGGGCCAAGGAA
TIM3	NM_032782.3	TATATGAAGTGGAGGAGCCCAATGAGTATTATTGCTATGTCAGCAGCAGGCAGCAACCCTCACAACCTTTGGGTTGTCGCTTTGCAATGCCATAGATCCA

Supplementary Table 2: 474 immune-related genes for volcano plot depiction

gene names									
ABCA1	CD14	DCBLD2	HLA-DOA	IGSF11	IRF9	MET	NRF1	PSMD8	SERPINB1
ABI1	CD163	DDX60	HLA-DPA1	IKZF1	IRGM	MIP1	NRK	PSMD9	SERPINB2
ACVR1	CD20	DHX58	HLA-DPB1	IL10	ISG15	MIP2	NSMCE2	PSPH	SERPINB3
ACVR1B	CD274	DOCK1	HLA-DQA1	IL10RA	ISG20	MIP3	OAS1	PTEN	SERPINB4
ACVR2A	CD276	DOCK2	HLA-DQB1	IL12A	ITGA1	MIP4	OAS2	PTPN1	SERPINB6
ADAM1	CD28	DPP10	HLA-DRA	IL12B	ITGA2	MIP5	OAS3	PTPN11	SERPINB8
ADGRE1	CD3E	DPP7	HLA-DRB1	IL13RA1	ITGA3	MIP6	OASL	PTPN2	SERPINC1
ADM	CD4	EBI3	HLA-DRB5	IL15	ITGA4	MMP1	OX40	PTPN22	SERPINE1
ADORA2B	CD40	EFEMP1	HLA-E	IL15RA	ITGA5	MMP2	OX40L	PTPN6	SIGLEC1
ADRM1	CD48	EIF2AK2	HLA-F	IL17A	ITGAV	MMP3	P2RY2	RAB5A	SIGLEC15
AHR	CD5	EIF4E3	HLA-G	IL17F	ITGB1	MMP7	P2RX4	RAB9	SIGLEC3
AIRE	CD55	ENG	HLA-H	IL17RA	ITGB2	MMP8	P2RX7	RAF1	SIGLEC6
ALCAM	CD56	ENO1	HLA-I HC	IL18	ITGB3	MMP9	P2RY14	RAP1A	SIGLEC9
ALOX5AP	CD69	ENOX1	HLA-HB	IL18BP	ITGB4	MS4A1	PARP1	RAP1B	SIGLECP1
AP2S1	CD70	EP300	HLA-KA	IL18R1	ITGB5	MS4A2	PARP12	RARA	SIPA1
APC	CD74	EPSTI1	HLA-LA	IL18RAP	ITGB6	MS4A3	PARP14	RARB	SLAMF1
APOL6	CD82	EREG	HLA-PLA	IL1A	ITGB7	MS4A4A	PARP3	RARG	SLAMF7
APLNR	CD86	ERI2	HLCS	IL1B	ITGB8	MS4A4E	PARP9	RB1	SLC11A1
ARID4B	CDKN1A	FAF1	HLTF	IL1F10	ITGBL1	MS4A6A	PAXIP1	RELA	SLC11A2
ARID5B	CDKN1C	FAS	HMBOX1	IL1R1	JAK1	MSR1	PDCD1	RELB	SLC2A1
ARL4A	CDKN2	FASLG	HMMR	IL20RB	JAK2	MVP	PDCD1LG2	RFXAP	SLC30A1
ASNS	CDKN2A	FES	HPGD	IL21	JAK3	NAMPT	PDGFRB	RFXANK	SLC38A1
ATP2A2	CDK9	FGFR2	HSD17B2	IL21R	KIF1B	NCOA3	PDE4B	RFXNP	SLC39A1
ATP2B1	CDMP2	FKBP1A	ICAM1	IL22RA1	KLF6	NCOR2	PIAS1	RIPK1	SLC39A3
ATP2C1	CDR3B	FLT3	ICOS	IL22RA2	KLRK1	NCR1	PIAS3	RIPK2	SLC39A4
AUTS2	CDX2	FOXP3	ICOSLG	IL23A	LAMP1	NDP	PIAS4	RORA	SLC4A1
AXIN1	CLEC5A	FPR1	ID1	IL23R	LAMP3	NFKB1	PIK3CA	RORC	SLC4A2
AXL	CFH	FYN	ID2	IL24	LCK	NFKB2	PIK3CB	RRAGD	SLC4A3
B2M	CIITA	GABBR1	ID3	IL2RB	LIF	NFKBIA	PIK3CD	RRNAD1	SLC4A4
BANK1	CLIC1	GCH1	IDO1	IL2RG	LILRB1	NFKBIE	PIK3R1	S100A1	SLC6A1
BATF2	CMPK2	GHR	IFI16	IL4	LILRB2	NFKBIZ	PIK3R2	S100A10	SLC7A1
BEST1	CMKLR1	GLB1	IFI35	IL4R	LILRB3	NLRP3	PIK3R3	S100A11	SLC7A11
BMP2	COL6A1	GNLY	IFI44	IL6	LILRB4	NMI	PIM1	S100A12	SLC7A2
BMP6	COL6A2	GPR18	IFI44L	IL6R	LILRB5	NOD1	PLA2G4A	S100A13	
BPGM	COL6A3	GPR132	IFIH1	IL7	LILRB6	NOD2	PLAUR	S100A2	
BST2	CX3CL1	GPR183	IFIT1	IL7R	LILRA1	NOX1	PML	S100A3	
BTG1	CXCL10	GPRC5A	IFIT2	IL8	LILRA2	NOX2	POMC	S100A4	
BTG2	CXCL11	GSTT1	IFIT3	IL9	LILRA3	NOX3	PPARA	S100A6	
CACNA1S	CXCL1	GZMA	IFITM1	IL9R	LILRA4	NOX4	PPARG	S100A7	
CCL1	CXCL2	GZMB	IFITM2	INDO	LILRA5	NOX5	PRKAA1	S100A8	
CCL17	CXCL3	HBEGF	IFITM3	INHBA	LILRA6	NR1H2	PRKAB1	S100A9	
CCL2	CXCL5	HDAC1	IFNA1	IRF1	LILRC1	NR1H3	PRKAB2	SAA1	
CCL20	CXCL6	HIF1A	IFNA2	IRF2	LILRC2	NR2C2	PRKCA	SAA2	
CCL22	CXCL8	HIPK2	IFNB1	IRF3	LRP1	NR3C1	PRKCQ	SAA4	
CCL24	CXCR3	HLA-A	IFNGR1	IRF4	LTA	NR4A1	PRKCH	SAMHD1	
CCL4L1	CXCR6	HLA-B	IFNGR2	IRF5	LYN	NR4A2	PRKCZ	SELENOS	
CCL5	CXCR7	HLA-C	IFNL1	IRF6	MARCO	NR4A3	PROKR2	SELE	
CCR7	CYBB	HLA-DMA	IFNL2	IRF7	MDM2	NRAS	PSMD5	SELL	
CCR8	CXXC1	HLA-DMB	IFNL3	IRF8	MDM4	NRBF2	PSMD6	SELP	

Supplementary Table 3: Overview of antibodies applied

Antibody	Clone	Supplier	Catalogue number	Dilution and antigen retrieval	Previous validation
CD3	SP7	Labvision, Germany	RM-9107-S	1:100, pH6	Validated for IHC, RUO
CD8	SP16	Abcam, UK	ab101500	1:100, pH6	Validated for IHC, RUO
CD69	EPR21814	Abcam, UK	ab233396	1:100, pH6	Validated for IHC, RUO
CD276	SP206	Abcam, UK	ab227670	1:100, pH6	Validated for IHC, RUO
CTLA4	Tinto CTLA-4/CD152	Medac, Germany	BSB 3484	RTU, pH9	Validated for IHC, IVD
CXCR4	UMB2	Abcam, UK	ab124824	1:300, pH9	Validated for IHC, RUO
ER	1D5	Thermo Scientific, MA, USA	MA5-13191	1:250, pH6	Validated for IHC, RUO
FoxP3	236A/E7	Abcam, UK	ab20034	1:100, pH9	Validated for IHC, RUO
Her2	DG44	DAKO, CA, USA	SK001	RTU, pH6	Validated for IHC, IVD
HLA-G	4H84	Abcam, UK	ab52455	1:200, pH6	Validated for IHC, RUO
IDO-1	V1NC3IDO	Thermo Scientific, MA, USA	14-9750-82	1:400, pH6	Validated for IHC, RUO
ISG-15	ab131119	Abcam, UK	ab131119	1:500, pH6	Validated for IHC, RUO
LAG3	12H6	Leica biosystems, Germany	PA0300-U	RTU, pH9	Validated for IHC, IVD
MHC class I HC	HC10	Thermo Scientific, MA, USA	MUB2037P	1:200, pH6	Validated for IHC, RUO
Ki67	SP6	Thermo Scientific, MA, USA	MA5-14520	1:100, pH6	Validated for IHC, IVD
Pan-CK	AE1/AE3 & 5D3	Zytomed, Germany	MSK098-05	1:200, pH9	Validated for IHC, RUO
PD-1	NAT105	Zytomed, Germany	NAT105	1:200, pH6	Validated for IHC, RUO
p-STAT1	Tyr701	Cell signaling, UK	# 9167	1:50, pH6	Validated for IHC, RUO
PR	PgR 636	DAKO, CA, USA	IR068	1:100, pH6	Validated for IHC, IVD
TAP1	EPR26236-92	Abcam, UK	ab322039	1:5000, pH9	Validated for IHC, RUO
