# Supplementary material for: HIV status alters immune cell infiltration and activation profile in women with breast cancer
Source: Nat Commun. 2025 May 20;16:4699. doi: 10.1038/s41467-025-59408-8 (PMC12092689; doi:10.1038/s41467-025-59408-8)
Supplement: Supplementary file 2 — Reporting Summary [file 41467_2025_59408_MOESM2_ESM.pdf]

nature portfolio

Corresponding author(s): *Double-anonymous peer review submissions: write DAPR and your manuscript number here instead of author names.*

Last updated by author(s): *YYYY-MM-DD*

Reporting Summary

Nature Portfolio wishes to improve the reproducibility of the work that we publish. This form provides structure for consistency and transparency in reporting. For further information on Nature Portfolio policies, see our Editorial Policies and the Editorial Policy Checklist.

Statistics

For all statistical analyses, confirm that the following items are present in the figure legend, table legend, main text, or Methods section.

n/a	Confirmed	
☐	☒	The exact sample size (*n*) for each experimental group/condition, given as a discrete number and unit of measurement
☐	☒	A statement on whether measurements were taken from distinct samples or whether the same sample was measured repeatedly
☐	☒	The statistical test(s) used AND whether they are one- or two-sided<br>*Only common tests should be described solely by name; describe more complex techniques in the Methods section.*
☐	☒	A description of all covariates tested
☐	☒	A description of any assumptions or corrections, such as tests of normality and adjustment for multiple comparisons
☐	☒	A full description of the statistical parameters including central tendency (e.g. means) or other basic estimates (e.g. regression coefficient) AND variation (e.g. standard deviation) or associated estimates of uncertainty (e.g. confidence intervals)
☐	☐	For null hypothesis testing, the test statistic (e.g. *F*, *t*, *r*) with confidence intervals, effect sizes, degrees of freedom and *P* value noted<br>*Give P values as exact values whenever suitable.*
☒	☐	For Bayesian analysis, information on the choice of priors and Markov chain Monte Carlo settings
☐	☒	For hierarchical and complex designs, identification of the appropriate level for tests and full reporting of outcomes
☐	☒	Estimates of effect sizes (e.g. Cohen's *d*, Pearson's *r*), indicating how they were calculated

Our web collection on statistics for biologists contains articles on many of the points above.

Software and code

Policy information about availability of computer code

Data collection	*Provide a description of all commercial, open source and custom code used to collect the data in this study, specifying the version used OR state that no software was used.*
Data analysis	Phython, GraphPad Prism, IBM SPSS

For manuscripts utilizing custom algorithms or software that are central to the research but not yet described in published literature, software must be made available to editors and reviewers. We strongly encourage code deposition in a community repository (e.g. GitHub). See the Nature Portfolio guidelines for submitting code & software for further information.

Data

Policy information about availability of data

All manuscripts must include a data availability statement. This statement should provide the following information, where applicable:

- Accession codes, unique identifiers, or web links for publicly available datasets
- A description of any restrictions on data availability
- For clinical datasets or third party data, please ensure that the statement adheres to our policy

Provide your data availability statement here.

Research involving human participants, their data, or biological material

Policy information about studies with human participants or human data. See also policy information about sex, gender (identity/presentation), and sexual orientation and race, ethnicity and racism.

Reporting on sex and gender	Only female BC patients were included in the study.
Reporting on race, ethnicity, or other socially relevant groupings	Only black patients from South Africa and Namibia were included in this study.
Population characteristics	Age, tumor stage and HIV status are given in the manuscript. Socio-economic data was included in multivariate analysis.
Recruitment	Patient samples were taken from ABC-DO and SACHO study cohorts.
Ethics oversight	Is given in Supplementary Table S1.<br>Martin Luther University, Halle-Wittenberg Germany 06/06/2014 2014-57<br>School of Anatomical Pathology, National Health Laboratory Services South Africa 27/07/2014 M140754<br>IARC France - IEC13-19, IEC15-18<br>Ministry of Health and Social Services of Namibia Namibia 2017 17/3/3<br>University of the Witwatersrand, Gauteng, South Africa - M150345

Note that full information on the approval of the study protocol must also be provided in the manuscript.

Field-specific reporting

Please select the one below that is the best fit for your research. If you are not sure, read the appropriate sections before making your selection.

[x] Life sciences [ ] Behavioural & social sciences [ ] Ecological, evolutionary & environmental sciences

For a reference copy of the document with all sections, see nature.com/documents/nr-reporting-summary-flat.pdf

Life sciences study design

All studies must disclose on these points even when the disclosure is negative.

Sample size	296 patients
Data exclusions	all samples were included in the analysis. Survival data was available for 291 patients.
Replication	#NA
Randomization	#NA
Blinding	#NA

Reporting for specific materials, systems and methods

We require information from authors about some types of materials, experimental systems and methods used in many studies. Here, indicate whether each material, system or method listed is relevant to your study. If you are not sure if a list item applies to your research, read the appropriate section before selecting a response.

Materials & experimental systems

n/a	Involved in the study	
[ ]	[x]	Antibodies
[x]	[ ]	Eukaryotic cell lines
[x]	[ ]	Palaeontology and archaeology
[x]	[ ]	Animals and other organisms
[ ]	[x]	Clinical data
[x]	[ ]	Dual use research of concern
[x]	[ ]	Plants

Methods

n/a	Involved in the study	
[x]	[ ]	ChIP-seq
[x]	[ ]	Flow cytometry
[x]	[ ]	MRI-based neuroimaging

Antibodies

Antibodies used	Antibody clone Supplier<br>CD3 SP7 Labvision, Germany<br>CD8 SP16 Abcam, UK<br>CD69 ab233396 Abcam, UK<br>CD276 ab227670 Abcam, UK<br>CTLA4 Tinto CTLA-4/CD152 Medac, Germany<br>CXCR4 UMB2 Abcam, UK<br>ER 1D5 Thermo Scientific, MA, USA<br>FoxP3 236A/E7 Abcam, UK<br>Her2 DG44 DAKO, CA, USA<br>HLA-G 4H84 Abcam, UK<br>IDO-1 V1NC3IDO Invitrogen, MA, USA<br>ISG-15 ab131119 Abcam, UK<br>LAG3 12H6 Leica biosystems, Germany<br>MHC class I HC HC10 Thermo Scientific, MA, USA<br>Ki67 SP6 Thermo Scientific, MA, USA<br>Pan-CK AE1/AE3 & 5D3 Zytomed, Germany<br>PD-1 NAT105 Zytomed, Germany<br>p-STAT1 Tyr701 Cell signaling, UK<br>PR PgR 636 DAKO, CA, USA<br>TAP1 ab13516 Abcam, UK
Validation	All primary antibodies were tested and validated using appropriate tissue samples.

Clinical data

Policy information about clinical studies
All manuscripts should comply with the ICMJEguidelines for publication of clinical research and a completedCONSORT checklist must be included with all submissions.

Clinical trial registration	#NA
Study protocol	#NA
Data collection	#NA
Outcomes	#NA

Plants

Seed stocks	#NA
Novel plant genotypes	#NA
Authentication	#NA
